# Does improvement management of atopic dermatitis influence the appearance of respiratory allergic diseases? A follow-up study

**DOI:** 10.1186/1476-7961-8-8

**Published:** 2010-06-30

**Authors:** Giampaolo Ricci, Annalisa Patrizi, Arianna Giannetti, Arianna Dondi, Barbara Bendandi, Massimo Masi

**Affiliations:** 1 Department of Pediatrics, University of Bologna, Italy; 2 Dermatology, Department of Internal Medicine, Geriatrics and Nephrologic Diseases, University of Bologna, Italy

## Abstract

**Background:**

Atopic dermatitis (AD) is often the prelude to allergic diseases. The aim of this study was 1) to evaluate if an integrated management regime could bring about a change in the evolution of the disease in comparison to the results of a previous study; 2) to determine whether the refinement of allergic investigations allowed to identify more promptly the risk factors of evolution into respiratory allergic diseases.

**Methods:**

The study included 176 children affected by AD and previously evaluated between 1993 and 2002 at the age of 9-16 months, who underwent a telephonic interview by means of a semi-structured, pre-formed questionnaire after a mean follow-up time of 8 years. According to the SCORAD, at first evaluation children had mild AD in 23% of cases, moderate in 62%, severe in 15%.

**Results:**

AD disappeared in 92 cases (52%), asthma appeared in 30 (17%) and rhinoconjunctivitis in 48 (27%). The factors significantly related to the appearance of asthma were: sensitization to food allergens with sIgE > 2 KU/L (cow's milk and hen's egg; *P *< 0.05); to inhalant allergens with sIgE > 0.35 KU/L (*P *< 0.05). Logistic regression analysis showed that inhalant sensitization was positively related to the occurrence of asthma (OR = 4.219). While AD showed similar rates of disappearance to those of our previous study, the incidence of asthma was reduced, at the same follow-up time, from 29% to 15% (*P *= 0.002), and the incidence of rhinoconjunctivitis from 35% to 24% (*P *= 0.02).

**Conclusion:**

Comparing the results with those of the previous study, integrated management of AD does not seem to influence its natural course. Nevertheless, the decrease in the percentage of children evolving towards respiratory allergic disease stresses the importance of early diagnosis and improvement management carried out by specialist centers. The presence of allergic sensitization at one year of age might predict the development of respiratory allergy.

## 

Atopic dermatitis (AD) is the most frequent chronic skin disease of childhood, with onset mainly in the first years of life. The prevalence of AD has doubled or tripled in industrialized countries over the past three decades: 15 to 30% of children and 2 to 10% of adults are affected [1]. In 70-80% of patients AD is associated with increased total serum IgE levels and food/inhalant specific (s) IgE levels, whereas in 20-30% there is no such sensitization [2].

In many cases, AD disappears or improves during childhood. However, in some cases the disease may persist into maturity and is associated with the development of asthma and/or allergic rhinitis. The risk of developing asthma in children with AD is highly variable: according to some authors the prevalence is 25% while others suggest higher values up to 80% [3-6]. This difference may be due to the use of different clinical and laboratory methods.

In a previous study [3] our team tried to assess the natural course of AD, as well as factors that affect its disappearance or persistence and the possible emergence of other allergic respiratory diseases. Children included in this study were aged between 6 and 36 months when they had their first visit between 1981 and 1989, involving the performance of allergometric tests and an assessment of the clinical picture and family history for atopy. After a follow-up of about 10 years, AD had disappeared completely in 124 cases (60.5%). Seventy children (34.1%) had developed asthma and 118 (57.6%) rhinoconjunctivitis (RC).

The main aim of this study was to determine whether a integrated clinical management had brought about a change in the evolution of the AD in comparison with the results of the previous study [3] carried out by our team in the preceding decade.

Furthermore, we wished to see if the refinement of clinical investigations (publication by the European Task Force of SCORAD index [7], 1993) and laboratory tests (determination of sIgE with quantitative method ImmunoCAP™, 1989) allowed us to identify more promptly the risk factors in children with AD and predict the evolution of AD into respiratory allergic diseases.

## Methods

### A. Study design

#### A.1. Phases of the study

This study consisted of two phases:

1) a retrospective analysis of children affected by AD at the age of 9-16 months;

2) telephone contact of the selected patients in order to evaluate the follow-up.

Only patients who were first evaluated, as infants, in our Pediatric Allergology Outpatients clinic were included, and the same team of physicians performed the follow-up telephone interviews.

It is interesting to note that, although our center may be considered a tertiary one, it is the practice of local national health pediatricians to send all patients with suspected AD, even with mild severity, to a specialist to perform allergometric assessment, so that the severity grading of this population has a wide variability.

#### A.2. Inclusion criteria

a) diagnosis of AD at an age of 9 to 16 months made at our Pediatric Allergology Outpatients Clinic, with a first clinical examination between 1993 and 2002;

b) availability of a detailed family and personal history;

c) performance of allergometric tests (skin prick tests (SPTs) and sIgE serum level for food and inhalant allergens);

d) telephone availability;

e) informed consent by the parents.

#### A.3. Exclusion criteria

1) patients who did not fulfill the inclusion criteria;

2) patients whose parents did not give their consent for inclusion;

3) patients who had other serious or invalidating associated pathologies.

### B. Clinical assessment and AD management

#### B.1. Clinical assessment

At the time of the first observation, the diagnosis of AD was made by the physicians on the basis of the criteria of Hanifin and Rajka [8]; parents or siblings were regarded as atopic if they reported a diagnosis of AD, RC or asthma.

At the first visit, the evaluation of the severity of AD was assessed by the SCORAD index [7].

SCORAD index < 25 shows a mild AD, 25-50 a moderate form, > 50 a severe form.

The new SCORAD index was not adopted to avoid affecting comparison with the previous study [3].

#### B.2. Management of AD

Since the '90s there has been an improvement in the global approach to children with AD both from an allergological and dermatological point of view. In particular an environmental prophylaxis has always has been recommended to all subjects positive to any allergens, along with a strict integrated management to obtain a very stringent control of flares with repeated control when SCORAD index is moderate or severe.

#### B.2.1. Environmental Prevention

Mite sensitization is often present in patients with atopic eczema, and may evoke or worsen skin reactions [9]. For children with sensitization to inhalant allergens, environmental prophylaxis was recommended in order to reduce the level of mites allergens in the home through bed encasing with special fabric textiles, the use of a high-filtration vacuum cleaning and the avoidance of objects containing dust in patient room.

#### B.2.2. Integrated management

The management of AD treatment has been widely discussed in literature [10] and in our previous work [11] and a step-based approach to the disease has been proposed. Dry skin should be only treated with emollients and avoidance of trigger factors. For mild to moderate and moderate to severe forms of AD, topical corticosteroids of increasing potency and/or topical calcineurin inhibitors are suggested. Systemic therapy should only be considered in case of severe, recalcitrant AD. When symptoms flare repeatedly, physicians may consider several systemic therapies, such as aggressive short-course systemic corticosteroids, immunosuppressants, biologicals, antimicrobials, anthistamines and leukotriene inhibitors.

#### B.3. Allergometric assessment

Allergometric assessment was performed at baseline (between 1993 and 2002) using SPT and determination of total and sIgE.

The determination of total serum IgE level was performed by PRIST (Pharmacia, Uppsala, Sweden); the value assumed as normal or increased was obtained by comparison with normal children of the same age group [12].

The determination of sIgE was performed by ImmunoCAP™ (Pharmacia, Sweden) and was measured in all patients for the following allergens: cow's milk, hen's egg, soybean, wheat, peanut, nut, codfish, apple, grass pollen, house dust mite (D. pteronyssinus, D. farinae), cat dander, dog dander. We considered as positive a sensitization to the allergen with a serum IgE level greater than 0.35 KU/L.

All the sera were tested for total and sIgE levels in the central laboratory of our hospital.

The SPT was made in all patients for the following allergens: cow's milk, hen's egg, soybean, wheat, peanut, nut, codfish, apple, grass pollen, house dust mite (D. pteronyssinus, D farinae), cat dander, dog dander, and Alternaria. Positivity was assessed by comparing the wheal of the allergens with that of the histamine, as suggested by the Consensus Conference of the Group of Allergology and Pediatric Immunology [13].

### C. Telephone interview

#### C.1. Contact with the families of children for the purposes of administering a phone questionnaire

**Figure 1:**
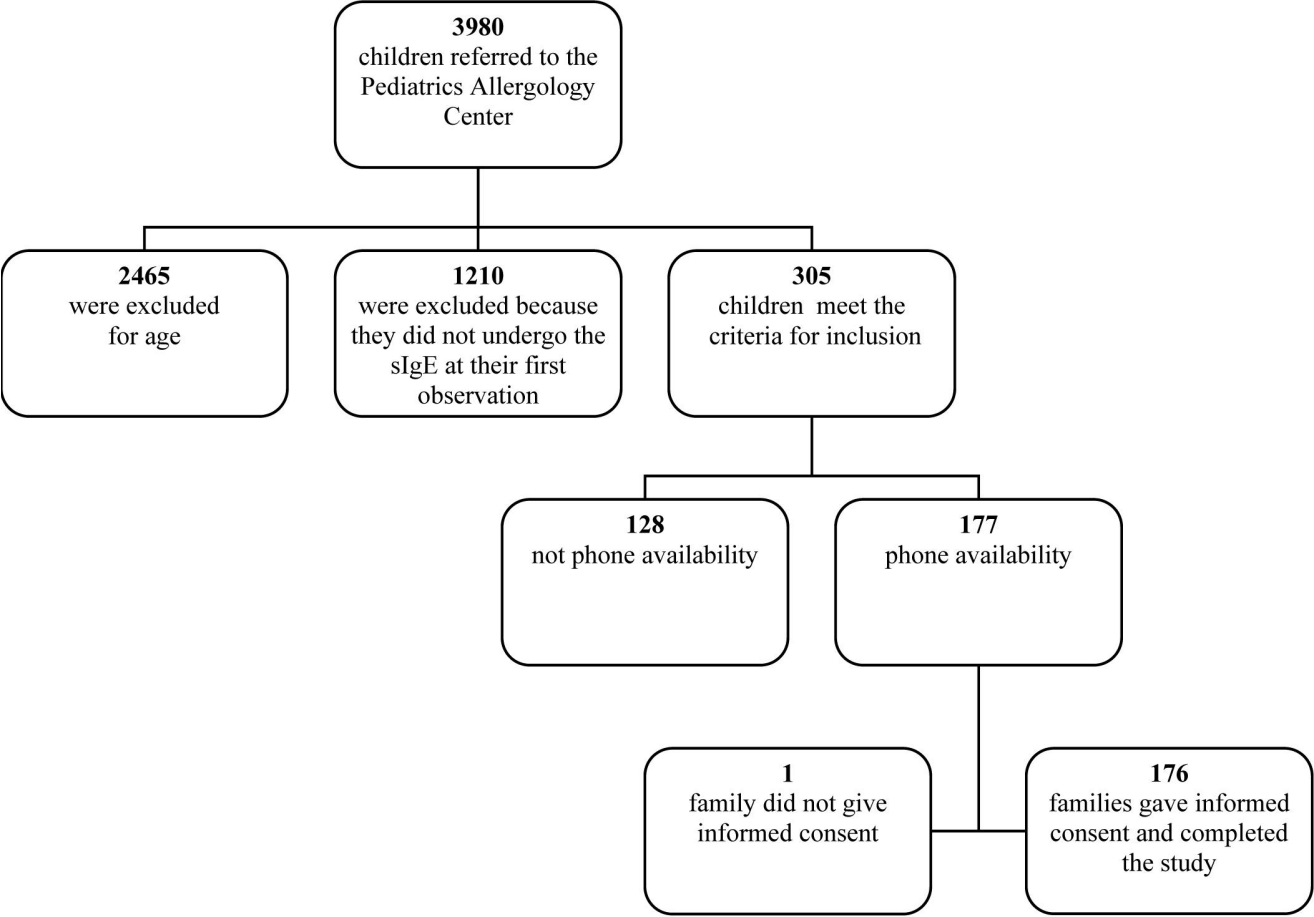
**Study population flow chart**.

Of the 177 selected children, 176 replied to the telephone questions (Fig.1). All the families were informed about the aim of the study and were generally very willing to supply the necessary information.

Parents of participants responded by telephon slightly modified versions of the International Study of Asthma and Allergies in Childhood (ISAAC) questionnaires [14].

Only the diagnoses of asthma and RC made by the general practitionnaire or by a pediatric allergologist/pneumologist on the basis of objective data were considered valid.

#### C.2. Availability for a check-up

During the telephone interview the possibility of a clinical allergologic evaluation was offered; this was welcomed by many parents (103 children agreed to follow up visit).

### D. Data collection and analysis

#### D.1. Completion of a database with the following information

- sex;

- age at first examination;

- assessment determination of the severity of the disease through a clinical score [7];

- SPT for food and inhalant allergens;

- total and sIgE serum level at first observation.

#### D.2. Statistical analysis

Elaboration of the data was made by an expert in statistics.

Statistical analysis was performed using software (SPSS 15 for Windows, SPSS Inc, Chicago, Ill). The evolution of AD was related to the following factors: sex, severity of the disease at first observation, family or personal history of first-degree atopy, sensitization to food and inhalant allergens (assessed by SPT and sIgE serum level), total IgE level, persistence and duration of AD, and appearance and age at onset of asthma and RC.

The presence or absence of the above-mentioned factors in patients with different severity scores of AD was evaluated by contingency table, whereas Fisher's exact test was used to test AD evolution for all the other mentioned variables that were dichotomous.

The duration of AD or onset age of RC and asthma were compared by Fisher's analysis of variance in the different severity classes of AD (with Scheffe's post hoc test if necessary), and by unpaired Student t test for all dichotomic variables. The risk of incomplete recovery of RC or asthma onset was evaluated by corrected odds ratio (OR) in models of logistic regression backward stepwise (likelihood) method. Type I error was accepted at P less than 0.05.

### E. Ethics

This study was only observational and did not interfere with the clinical management of the patients, so it was not submitted to the ethical committee for approval. However, both the parents and the patients were informed that the questionnaire was proposed in an experimental manner; they were given the questionnaire only after obtaining an informed consent.

## Results

A total of 177 children fulfilling the above-mentioned criteria of inclusion were contacted (Fig.1). The interview questionnaire was completed for 176 children (Fig.1.) (98 boys, 56%, and 78 girls, 44%) (99.4%). The mean follow-up of these patients was 91.3 ± 24.2 months (range 6-12 years).

The mean age of children at first observation was 11.7 months. At follow-up the mean age was 102.8 months.

### Baseline data

#### Familial atopy

The evaluation of the first-degree familial atopy was possible only for 175 children because one child had been adopted. In 58 patients (33%) the father was atopic, in 62 (35%) the mother and in 27 (15%) one or more brothers or sisters.

#### Severity of AD

At first examination 40 of the 176 children (23%) had mild AD (SCORAD index < 25), 110 (62%) moderate AD (SCORAD index between 25 and 50 ), 26 (15%) severe AD (SCORAD index > 50).

#### Total IgE serum level

Total IgE serum level was increased in 89 cases (51%) out of the 174 (the IgE value of two children was not available) without any significant difference for sex.

The group of 85 children with normal IgE had a positive family history for atopy in 51 cases (60%).

The group with increased IgE had a positive family history for atopy in 57 cases out of 88 (65%).

The severity of AD was related to the values of the total IgE serum level at the first examination, with no statistical significance, even though in the severe forms of AD increased IgE levels were present in 17 cases out of 26 (65%).

#### SPT

One hundred patients out of 176 (57%), showed a sensitization to the SPT for at least one of the tested foods and/or inhalants.

Positivity for SPT did not show any significant difference between the group of children with normal IgE (54%) and that with increased IgE (58%).

Ninety-seven children were positive for food allergens and 13 positive for inhalant allergens; of these, 3 children were positive only for inhalants.

#### Serum sIgE

One hundred and three out of 176 patients (58.5%) had elevated levels of sIgE for at least one allergen at first observation.

The positive subjects were subsequently divided into two groups: the first (44 cases) contained children with sIgE level between 0.35 KU/L and 2 KU/L, the second (59 cases), subjects with a value > 2 KU/L. This cut-off value was decided upon the work by Nickel and colleagues [12].

### Data noted at follow-up

#### Outcome and evolution of AD

After the mean seven and a half year follow-up AD was still present in 84 children (48%) out of 176, in 27 cases (15%) with a persistent course, in 57 cases (32%) intermittent, while in 92 cases (52%) AD had disappeared.

Of the children still suffering from AD, 37 out of 84 (44%) had a single location, primarily on the limbs, while 15 out of 84 (18%) had multiple locations.

The mean age of disappearance of the AD was 3.25 years. The mean age of disappearance was slightly higher in severe AD (3.8 ± 1.0 years) than in the moderate (3.4 ± 0.4 years) or mild (2.8 ± 0.6 years) forms, but no statistically significant difference was found. The same trend coud be seen in children with hen's egg sensitization: a longer period was required in severe AD (3.7 ± 1.5 years) than in moderate (3.1 ± 0.6 years) or mild (2.4 ± 0.6 years) forms but also in this case the difference was not significant.

#### Onset of respiratory allergic diseases

Respiratory allergic diseases appeared in 66 cases out of 176 (37.5%).

Of the 66 cases, 36 patients out of 176 (20.5%) developed only RC, 18 out of 176 (10%) developed only asthma and 12 children out of 176 (7%) developed both RC and asthma.

#### 1) Allergic Rhinoconjunctivitis

The mean age of appearance of RC was 4.83 years.

In the 48 patients affected, the RC, both alone and in association with asthma, had a seasonal course in 41 cases (85.5%), whereas in 7 cases (15%) it was perennial.

#### 2) Asthma

The mean age of appearance of asthma was 3.33 years.

Out of 66 cases with respiratory allergic diseases, 30 (17%) had asthma, which in 18 cases was isolated and in 12 associated with RC.

All of the participants suffering from asthma had undergone permanent or transitory therapy during the last year: inhalant steroids in 25 cases (83%), short acting beta2 agonists in 14 cases (47%), long acting in 8 cases (27%) and antileukotrienes in 7 cases (23%).

In 26 cases out of 30 (87%) the asthma was under control (on the basis of the replies to the telephone questionnaire); in 4 cases the disease was only partially under control.

Seventeen out of the 18 patients with isolated asthma had their IgE serum level evaluated at first observation: 7 had normal IgE and 10 had increased IgE.

The total IgE serum level at first observation was significantly correlated with the appearance of the asthma, both isolated and in association with RC (*P *< 0.05). In fact the children with elevated levels of total IgE serum level at baseline showed a relative risk of developing asthma that is two times higher than those with normal levels.

Besides, the children with elevated levels of total IgE serum level at baseline showed a relative risk of developing asthma in association with RC that is four times greater in comparison to those with normal levels.

In the 30 patients with asthma, both isolated and associated with RC, these disorders were correlated with seasonal allergens in 22 cases (73%), and with perennial allergens in 8 cases (27%).

Of the 30 children suffering from asthma, 20 presented intermittent forms and 11 of these (55%) had increased IgE; 6 cases had a mild persistent asthma with increased IgE. The severe persistent asthma was present in 2 cases (one with IgE serum level increased at baseline).

#### Comment at follow-up

During the follow-up AD had disappeared in 92 children (52%) but if we also include the absence of respiratory complications only 54 children (30.7%) showed no symptoms.

**Figure 2:**
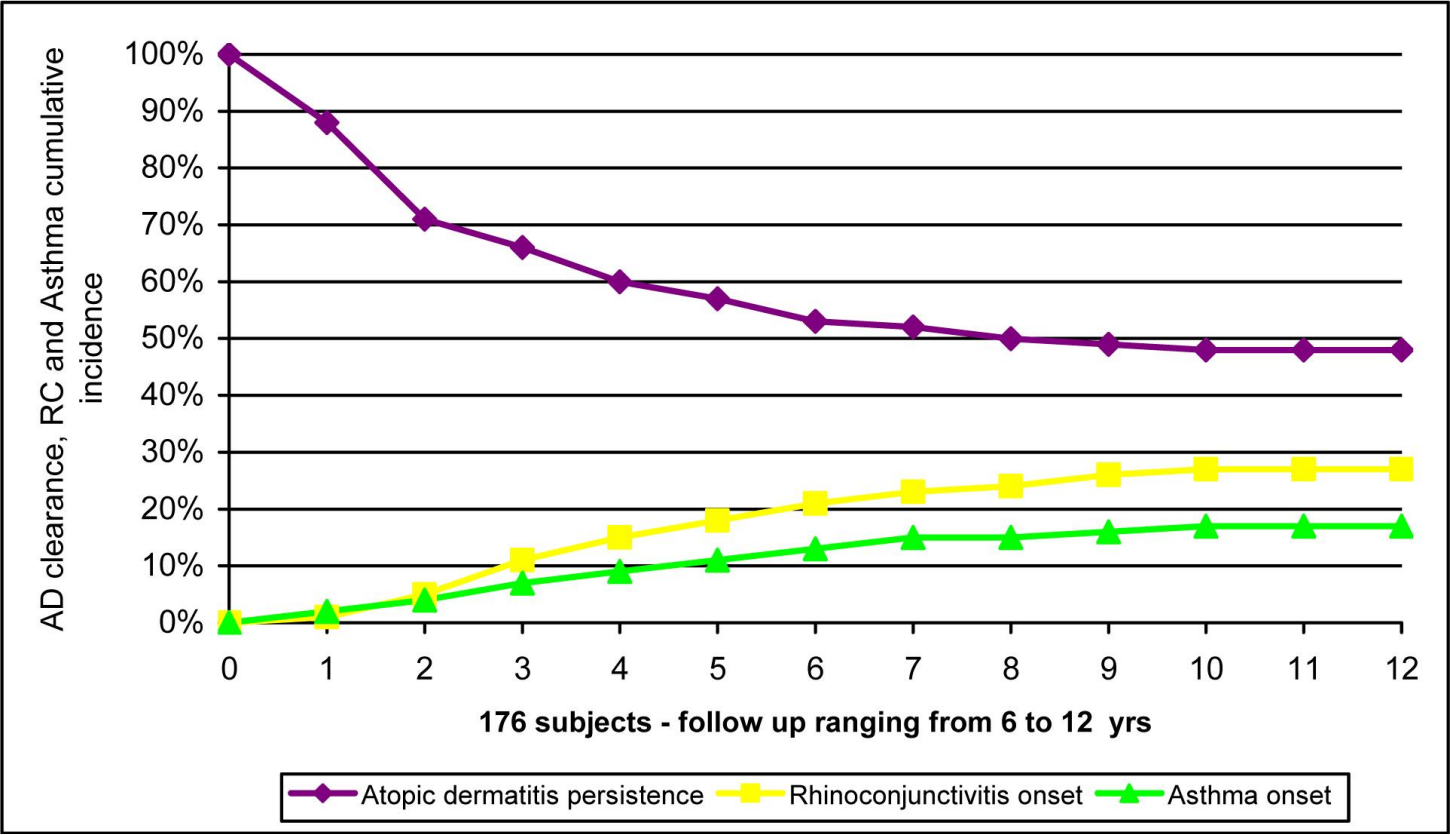
**Curves related to the persistence of AD and to the appearance of RC, asthma, in the 176 children participating in the study**.

RC had appeared in 48 children (27%), asthma in 30 (17%) (Fig.2).

**Figure 3:**
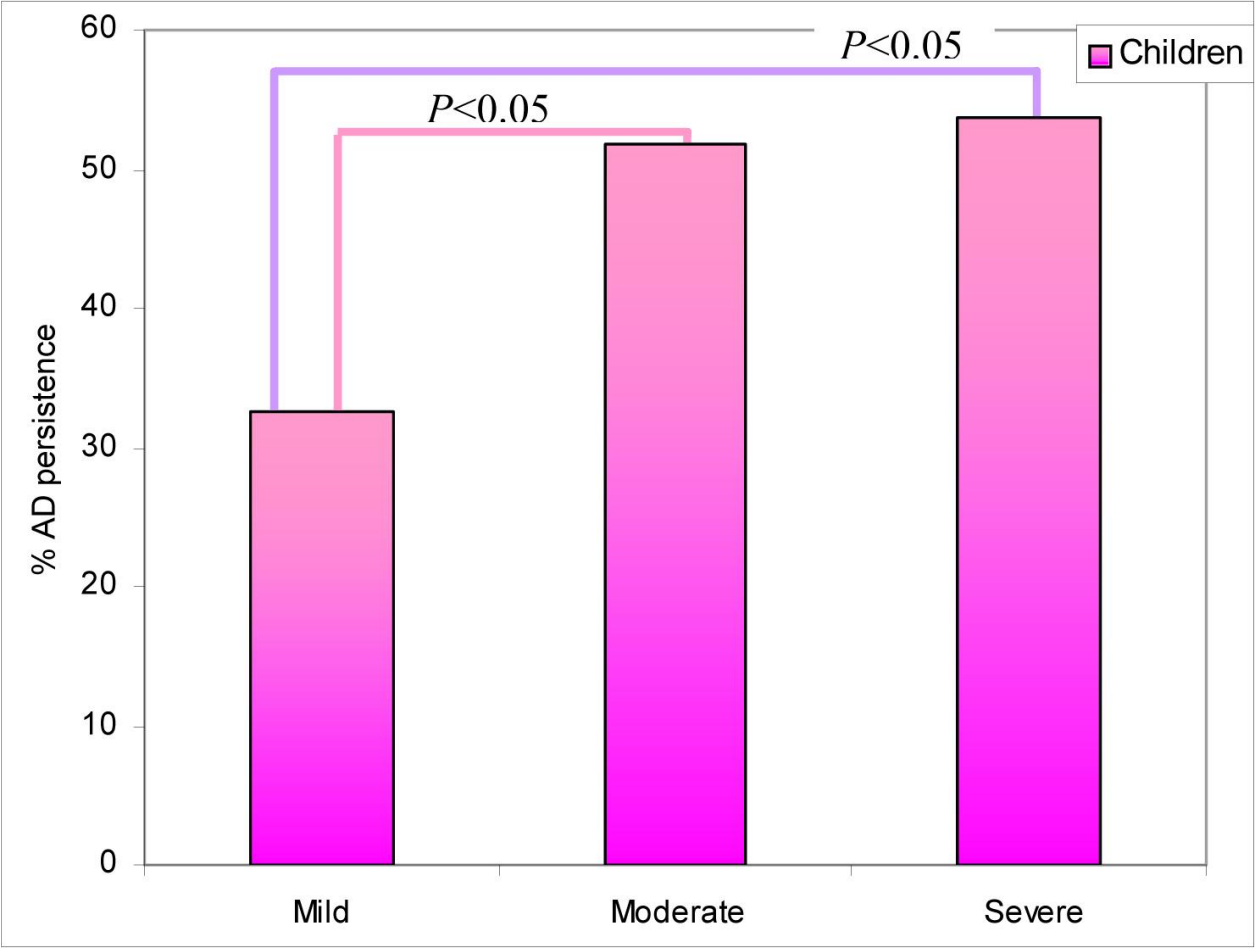
**Persistence of AD in relation to the severity of the AD at first observation**.

In the following figure (3) and table 1, table 2 and table 3) the different variables are considered in relation to the evolution of AD.

**Table 1:** Persistence of AD and appearance of RC and/or asthma in relation to food sensitization and inhalant sensitization assessed through sIgE (levels of IgE > 0.35 KU/L, between 0.35 and 2 KU/L and > 2 KU/L), and through SPT at first observation.

Variables	Category	N	AD persistence	RC appearance	Asthma appearance
Food sensitization	Absent	73	30	21	9
(specific IgE > 0.35 KU/L)			41%(29.8-52.4)	29%(18.4-39.2)	12%(4.8-19.9)

	Present	103	54	27	21
			52%(42.8-62.1)	26%(17.7-34.7)	20%(12.6-38.2)

Food sensitization	Absent	130	57	39	24
(specific IgE 0.35-2 KU/L)			44%(35,3-52,4)	30%(22,1-37,9)	18%(11,8-25,1)

	Present	46	27	9	6
			59%(44,5-72,9)	20%(8,1-31,0)	13%(3,3-22,8)

Food sensitization	Absent	119	57	30	15
(specific IgE > 2 KU/L)			48%(38.9-56.9)	25%(17.4-33.0)	13%(6.6-18.6)$

	Present	57	27	18	15
			47%(34.4-60.3)	32%(18.5-43.6)	26%(14.9-37.7)$

Food sensitization	Absent	79	33	22	6
(PRICK)			41.8%(30.9-52.6)	27.8%(18.0-37.7)	7.6%(1.8-13.4)£

	Present	97	51	26	24
			52.6%(42.6-62.5)	26.8%(18.0-35.6)	24.7%(16.2-33.3)£

Inhalant sensitization	Absent	140	62	37	19
(specific IgE > 0.35 KU/L)			44%(36,1-52,5)	26%(19,1-33,7)	14%(7,9-19,9)$

	Present	36	22	11	11
			61%(45,2-77,0)	31%(15,5-45,6)	31%(15,5-45,6)$

Inhalant sensitization	Absent	163	79	41	27
(PRICK)			48.5%(40.8-56.1)	25.2%(18.5-31.8)$	16.6%(10.9-22.3)

	Present	13	5	7	3
			38.5%(12.0-64.9)	53.8%(26.7-80.9)$	23.1%(0.2-46.0)

Confidence intervals 95% for each item.

Atopic Dermatitis (AD), Rhinoconjunctivitis (RC), Immunoglobulin E (IgE).

$ Fisher's exact test; *P *< 0.05

£ Fisher's exact test; *P *< 0.005

**Table 2:** Persistence of AD and appearance of RC and/or asthma in relation to cow's milk sensitization assessed through sIgE (levels of IgE > 0.35 KU/L, between 0.35 and 2 KU/L and > 2 KU/L), and through SPT at first observation.

Variables	Category	N	AD persistence	RC appearance	Asthma appearance
Milk sensitization	Absent	102	46	28	13
(specific IgE > 0.35 KU/L)			45,1%(35,4-54,8)	27,5%(18,8-36,1)	12,7%(6,3-19,2)

	Present	74	38	20	17
			51,4%(40,0-62,7)	27,0%(16,9-37,1)	23,0%(13,4-32,6)

Milk sensitization	Absent	137	62	38	24
(specific IgE 0.35-2 KU/L)			45%(36.9-53.6)	28%(20.2-35.2)	18%(11.2-23.9)

	Present	39	22	10	6
			56%(40.8-72.0)	26%(11.9-39.3)	15%(4.1-26.7)

Milk sensitization	Absent	141	68	38	19
(specific IgE > 2 KU/L)			48%(40.0-56.5)	27%(19.6-34.3)	13%(7.8-19.1)$

	Present	35	16	10	11
			46%(29.2-62.2)	29%(13.6-43.5)	31%(16.0-46.8)$

Milk sensitization	Absent	128	56	35	16
(PRICK)			43.8%(35.2-52.3)	27.3%(19.6-35.1)	12.5%(6.8-18.2)&

	Present	48	28	13	14
			58.3%(44.4-72.3)	27.1%(14.5-39.7)	29.2%(16.3-42.0)&

Confidence intervals 95% for each item.

Atopic Dermatitis (AD), Rhinoconjunctivitis (RC), Immunoglobulin E (IgE).

$ Fisher's exact test; *P *< 0.05

& Fisher's exact test; *P *< 0.01

**Table 3:** Persistence of AD and appearance of RC and/or asthma in relation to hen's egg sensitization assessed through sIgE (levels of IgE > 0.35 KU/L, between 0.35 and 2 KU/L and > 2 KU/L), and through SPT at first observation.

Variables	Category	N	AD persistence	RC appearance	Asthma appearance
Hen's egg sensitization	Absent	97	42	27	12
(specific IgE > 0.35 KU/L)			43,3%(33,4-53,2)	27%(18.9-36,8)	12,4%(5,8-18,9)

	Present	79	42	21	18
			53,2%(42,2-64,2)	26,6%(16,8-36,3)	22,8%(13,5-32,0)

Hen's egg sensitization	Absent	145	68	43	25
(specific IgE 0,35-2 KU/L)			47%(38,8-55,0)	30%(32,2-37,1)	17%(11,1-23,4)

	Present	31	16	5	5
			52%(34,0-69,2)	16%(3,2-29,1)	13%(1,1-24,7)

Hen's egg sensitization	Absent	128	58	32	17
(specific IgE > 2 KU/L)			58%(36.7-53.9)	25%(17.5-32.5)	13%(7.4-19.2)$

	Present	48	26	16	13
			54%(40.1-68.3)	33%(20.0-46.7)	27%(14.5-39.7)$

Hen's egg sensitization	Absent	97	42	27	11
(PRICK)			43.3%(33.4-53.2)	27.8%(18.9-36.8)	11.3%(5.0-17.7)$

	Present	79	42	21	19
			53.2%(42.2-64.2)	26.6%(16.8-36.3)	24.1%(14.6-33.5)$

Confidence intervals 95% for each item.

Atopic Dermatitis (AD), Rhinoconjunctivitis (RC), Immunoglobulin E (IgE).

$ Fisher's exact test; *P *< 0.05

A logistic regression backward stepwise (likelihood ratio) method was performed for evaluation of AD persistence, RC and asthma onset. The most suited models are expressed in Table 4. The children that developed RC (OR = 2.616), or were positive to at least one inhalant (with sIgE > 0.35 KU/L at first observation) (OR = 4.219) showed a greater risk of developing asthma.

**Table 4:** Evolution of AD in 176 children: retrospective analysis aimed at identifying the associated risk factors linked to the appearance of RC and asthma, made through the evaluation of the odds correct ratio (OR) of models of logistic regression applied to the data.

Dependent variables	Covariate	OR	OR LCL 95%	OR UCL 95%	P
**RC onset**	Asthma onset	2.764	1.108	6.896	0.029
	Female	0.461	0.212	1.001	0.050
**Asthma onset**	RC onset	2.616	1.034	6.617	0.042
	Inhalant sensitization	4.219	1.191	14.948	0.026

Logistic regression applied to characterize risk factors or protective factors from asthma and RC.

Atopic dermatitis (AD); lower confidence limit (LCL); odds ratio (OR); rhinoconjunctivitis (RC); upper confidence limit (UCL).

## Discussion

Regarding the clinical course of AD, the percentages of persistence are extremely variable in the literature, ranging from 8-13% to 60-70% [15-18]. Percentages of healing with age in the literature are also variable. Some [19] state 50-70% healing by the age of 10 years, others [20] 43.2% by the age of 3 and some [20,21] refer about a general improvement in AD severity by the fith-seventh year of life, with estimates of only 16% of persistence of AD after adolescence [22].

The discrepancy between these data can be partly explained by the fact that the methods used for diagnosis (the criteria of Hanifin and Rajka [8] since the eighties) and for the evaluation of severity of AD (SCORAD index [7] introduced since 1993) were different in the various series of cases. For these reasons we selected children born in or after 1993, when we started applying the aforementioned criteria. In the 8 year follow-up of the present study, 50% of the children still had AD. Our data suggest that, among all the various parameters, the greatest influence on the persistence of AD is the severity of the disease estimated through the SCORAD index [7] at the moment of the first observation (Pearson χ^2 ^= 4.846; *P *< 0.05) (Fig.3).

In our study, the mean age of disappearance is slightly higher in severe AD (3.8 ± 1.0 years) than in the moderate (3.4 ± 0.4 years) or mild (2.8 ± 0.6 years) forms, but no statistically significant difference was found. The same trend can be seen in children with hen's egg sensitization: a longer period is required in severe AD (3.7 ± 1.5 years) than in moderate (3.1 ± 0.6 years) or mild (2.4 ± 0.6 years) forms but also in this case there is no significant difference.

In a previous work [3], our team tried to assess the natural course of AD, as well as factors that affect the disappearance or persistence and the possible emergence of other allergic respiratory diseases. Children included in this study were aged between 6 and 36 months at the time of their first visit (between 1981 and 1989) and had carried out allergometric tests and an assessment of the clinical picture and family history for atopy. Concerning AD, the clinical characteristics of that population were similar to those of the population of the present study: more patients had mild AD (33% vs 23% in the present study) and less had moderate AD (48% vs 62%), but the frequency of severe AD was more or less the same (19% vs 15%). The population of the previous study had a lower prevalence of sensitization to food allergens (37% vs 58%) and a higher prevalence of sensitization to inhalant allergens (26% vs 20%), and this might be explained by the age at first evaluation, which was higher (6 to 36 months, compared to 9 to 16 months of the present study). After a follow-up of about 10 years, we reported that AD persisted in 39.5% of cases and the mean age of disappearance was 5.6 years.

Many data point towards a strong correlation between AD in early infancy and the subsequent appearance of asthma [23]. Children with AD present an increased frequency of RC and asthma, with percentages varying from 25% to 80% [3-6]. Therefore, identifying subjects at risk of complications in pediatric age could help prevent its onset.

In the present study the percentages of appearance of respiratory allergies is lower; in fact only 37.5% of children with AD developed respiratory pathologies, RC in 27% of cases and asthma alone in 17%. This difference can be explained partly by our lower degree of severity of AD, and partly by non homogeneous diagnostic criteria. In fact in several of the studies previously quoted the diagnosis of AD was made using criteria not standardized and not comparable until the eighties when the criteria of Hanifin and Rajka [8] were published, criteria also adopted in this study. This could have resulted in some differences in the clinical evaluation. The incidence of asthma in our case study is not high, perhaps partly explainable by the fact that we considered as asthmatics only the patients with a medical diagnosis of asthma, excluding the episodes of "wheezing" in the first years of life.

**Figure 4:**
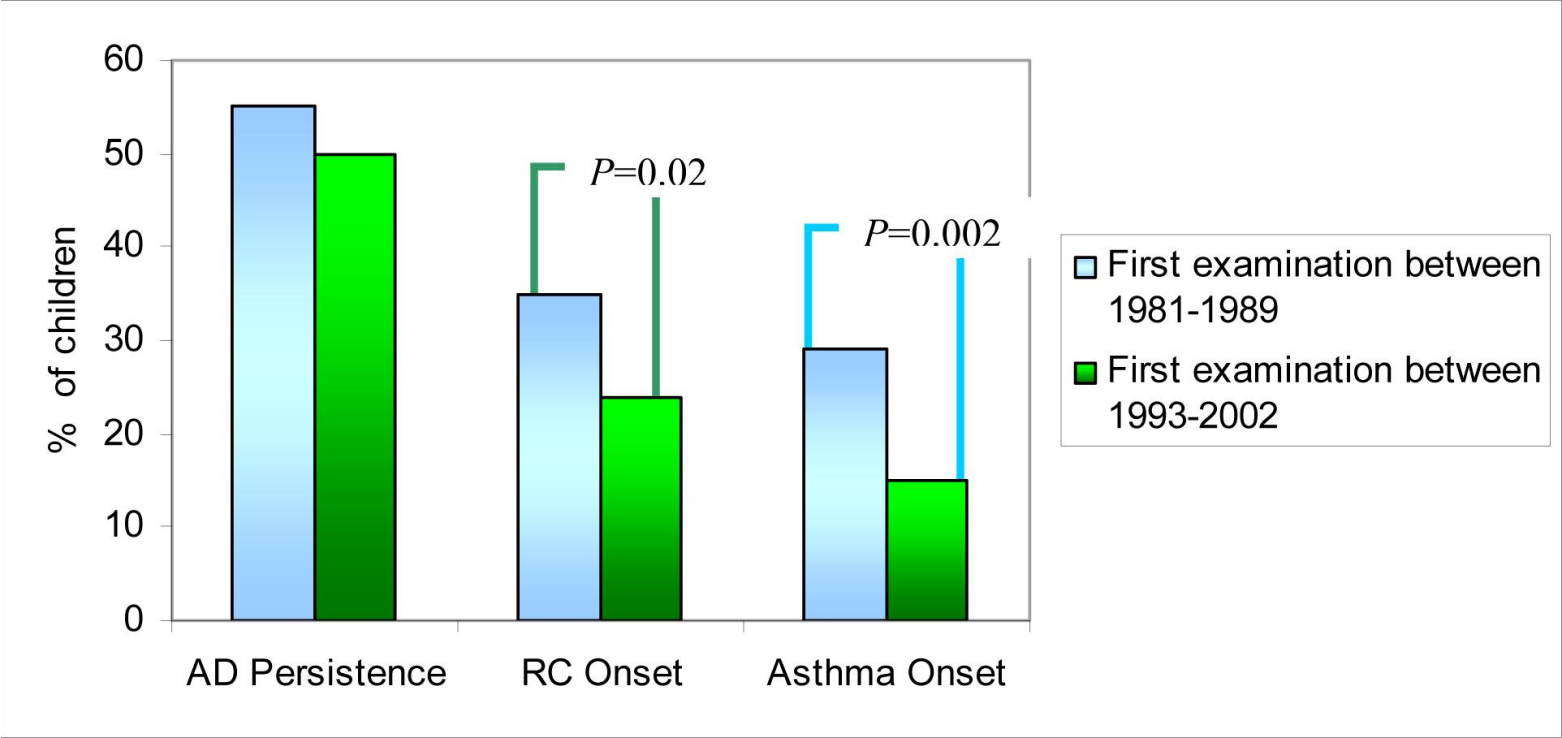
**Persistence of AD and appearance of RC, asthma in comparison with previous study **[3]** at the same follow-up time (8 years) (Previous study **[3]**: AD persistence: 113/205, RC onset: 72/205, Asthma onset: 59/205; Present study: AD persistence: 88/176, RC onset: 42/176, Asthma onset: 26/176)**.

In the aforementioned previous study by our group [3], involving children that had been first evaluated between 1981 and 1989, with similar characteristics, 57.6% of patients developed RC and 34.1% asthma. Furthermore, in our two studies the onset of these pathologies in the majority of cases occurred before puberty (Fig.2). The curve of appearance of asthma (Fig.2) reveals that at 8 years of age (mean age of our follow-up) the presence of asthma in our previous study [3] was already around 29%, while in the present study it is about 15% (Pearson χ^2 ^= 9.928 and *P *= 0.002) (Fig.4); a reduction in the appearance of asthma was therefore noted. Also the percentage of RC had fallen from 35% to 24% (Pearson χ^2 ^= 5.200 and *P *= 0.023) (Fig.4). This better result could be related to an improvement in the clinical management of AD. However, only follow-up will tell whether this trend is confirmed or asthma appearance is only postponed.

Van Der Hulst et al. [24] recently made a systematic review to analyze the risk for a child that has had AD in the first 4 years of life of developing asthma later in childhood. The review showed that after a varying period of follow-up, the prevalence of asthma varied from 33.7% to 52.5% in inpatients and outpatients respectively, and from 14.2% to 45.5% in the mixed group.

In the work of Oshshima et al. [25], the persistence of AD is strongly correlated with the onset of asthma. Gustaffson et al. [26], in an assessment of 92 children (aged 4-35 months) with AD after an 8 year follow-up, show that 70% of patients with a severe form developed asthma, compared to only 30% of children with a mild form.

Also in our previous study [3] we observed that a high SCORAD index at the first observation is associated with a greater probability of appearance of asthma (Pearson χ^2^= 14.225 and *P *< 0.007). On the other hand, the results of the present study do not show any statistical significance, even though it is possible to note a tendency pointing to a relationship between AD and asthma; 15% of children with the light form developed asthma, compared to 15.5% of those with moderate form and 26.9% of those with the severe form. It is possible once again that these statistics are influenced by the fact that there were few children suffering from severe dermatitis and a more limited cohort.

In a study by Schafer et al. [27] involving 2201 children aged between 5 and 14 years, the severity of AD was again correlated to a greater probability of developing RC.

Parental atopy is a well documented risk factor for the evolution of AD into allergic respiratory disease [25,28]. Our results confirm this association, since 21.8% (Fisher's exact test; *P *< 0.05) of children with first-degree familial atopy developed asthma.

Elevated levels of total IgE have been correlated with the risk of developing asthma by Burrows [29] and Wuthrich [30]. This also emerged from our study: 22.5% of children with increased IgE developed asthma in comparison to 10.6% with normal IgE (Fisher's exact test; *P *< 0.005). These children appear to have a relative risk of developing asthma that is twice that of those with normal levels of IgE (RR = 2.12).

The aim of this work is to verify whether the refinement of the allergic investigations (in 1989 the determination of sIgE changed, passing from a semiquantitative method to a quantitative method) had allowed us to identify more quickly the risk factors of AD evolving into respiratory allergic diseases and to improve the course of the disease.

In the literature there appears to be no reports focusing on long-term studies of clinical and allergometric evaluations observed during the course of AD with respect to its evolution and association with allergic responses in affected patients. The presence of an allergic sensitization (foods and/or inhalnts) is an important risk factor for the evolution into respiratory allergic disease [31]. Tables 1, 2 and 3 show that the atopic sensitization assessed both through sIgE serum levels and SPT is significantly correlated to the appearance of asthma. Moreover, the evaluation of sIgE with the quantitative method allowed us to verify that some positivities are particularly significant, such as that linked to atopic sensitization for hen's egg and cow's milk (unlike in our previous work [3]) with values of sIgE > 2 KU/L (Tab. 2 and 3). Our results are in accordance with those of Nickel [12] where the sensitization to hen's egg at one year of age seems to be predictive of a sensitization to inhalant allergens in late infancy. This is also confirmed by a literature [25,32]. The observation of a relationship between sensitization to foods and inhalants, and the subsequent appearance of asthma suggests that this group of children is particularly at risk and could be included in a program of preventive intervention.

One of the limitations of our study is the lack of some demographic data of children, including immigration status, parents' level of education, family income. These are all factors that might influence the persistence or remission of atopic dermatitis. Another limitation is due to the possible selection bias linked to disease severity: children with more severe disease might have been more easily contacted because of more recent routine follow-up visits, as compared to children with less severe disease. However, this possible bias might have influenced also our previous study and should not affect the final result and the comparison of the emerging data.

In conclusion, our work highlighted the importance of the adoption of both a quantitative evaluation of sIgE and improved clinical tools for the assessment of AD (SCORAD index, environmental prevention, integrated management) in the routine clinical practice. Sensitization to milk and hen's egg with values of sIgE > 2KU/L can be included among the risk factors for the development of asthma. Positivity to inhalants, on the other hand, is already a risk factor at values > 0.35 KU/L at one year of life. Although there appears to be a strong association between AD and allergy, the natural course of the disease seems poorly related to the allergic sensitization. However, given the decrease in the percentage of children evolving toward respiratory allergic disease compared to previous study [3], we stress the importance of early diagnosis, of therapeutic measures and of assiduous clinical check-ups carried out by specialist centers. Finally, our study has also highlighted the validity of the SPT in childhood as a means of early detection of subjects susceptible to respiratory allergic diseases.

## Competing interests

The authors declare that they have no competing interests.

## Authors' contributions

GR: planning, study design and manuscript preparation.

AP and MM: supervision and manuscript preparation.

AG: data collection, data analysis, literature search, abstract presentation, manuscript preparation.

AD: data collection, data analysis, literature search.

BB: data analysis, literature search.

All authors have read and approved the final manuscript.
